# Case of Rupture of the Uterus, and Recovery

**Published:** 1856-01

**Authors:** 


*Case of Rupture of the Uterus, and Recovery.* By W. W. DUVALL, M.D., of George's County, Maryland.

June 8, 1854, I was called to see —— ——, who had been in labor 12 hours; but, for four hours previous to my visit, there had been an entire suspension of uterine effort. Upon examination, the shoulder was found presenting. Turning the child, delivery by the feet was resorted to and effected with but little difficulty and delay—the child being dead. The uterus being passive the placenta was retained, and as there was considerable hæmorrhage, its extraction was deemed necessary, which was done—it being detached from the uterus, and lying near its mouth. The hæmorrhage not ceasing, or abating, so far as to render the patient's condition one of safety, it was thought advisable to introduce the hand to provoke contraction, and upon so doing, I perceived a transverse rent in the walls of the uterus, about three inches above the cervix, anteriorly, through which I could easily pass my index, middle, and ring finger. The patient being much exhausted, a neat and efficient bandage was applied around the abdomen. She was enjoined to lie upon her back, and opiates and cordials were administered. The lochial flux was excessive for several days, followed by sero-sanguineous and then purulent discharge, which continued for several weeks, accompanied by irritative fever and diarrhœa. The patient had borne three children previously, and the presumption is that the laceration occurred by the violent and unavailing efforts of the organ under the malpresentation, as there was but slight effort required in turning the child. Since her recovery, she has enjoyed good health, and menstruating regularly — having lived absque marito.—*The American Journal of the Medical Sciences.*

